# A survey of accepted authors in computer systems conferences

**DOI:** 10.7717/peerj-cs.299

**Published:** 2020-09-28

**Authors:** Eitan Frachtenberg, Noah Koster

**Affiliations:** Department of Computer Science, Reed College, Portland, OR, United States of America

**Keywords:** Computer Systems, Author survey, Researcher Diversity, Peer Review

## Abstract

Computer Science researchers rely on peer-reviewed conferences to publish their work and to receive feedback. The impact of these peer-reviewed papers on researchers’ careers can hardly be overstated. Yet conference organizers can make inconsistent choices for their review process, even in the same subfield. These choices are rarely reviewed critically, and when they are, the emphasis centers on the effects on the technical program, not the authors. In particular, the effects of conference policies on author experience and diversity are still not well understood. To help address this knowledge gap, this paper presents a cross-sectional study of 56 conferences from one large subfield of computer science, namely computer systems. We introduce a large author survey (*n* = 918), representing 809 unique papers. The goal of this paper is to expose this data and present an initial analysis of its findings. We primarily focus on quantitative comparisons between different survey questions and comparisons to external information we collected on author demographics, conference policies, and paper statistics. Another focal point of this study is author diversity. We found poor balance in the gender and geographical distributions of authors, but a more balanced spread across sector, experience, and English proficiency. For the most part, women and nonnative English speakers exhibit no differences in their experience of the peer-review process, suggesting no specific evidence of bias against these accepted authors. We also found strong support for author rebuttal to reviewers’ comments, especially among students and less experienced researchers.

## Introduction

Peer review is a cornerstone of modern scientific research. However, understanding and improving this process is challenging because it can be hard to experiment with peer review  (Beverly & Allman, 2013; Ernst & Resch, 1994; Mahoney, 1977; McNutt et al., 1990). For example, reputable conferences disallow parallel submissions, and even within the same conference, we cannot design an experiment where papers are reviewed multiple times with fully controlled variations. Perhaps the closest a study came to being a controlled experiment recently was a study on the NIPS 2014 conference, which found high inconsistency in the review outcomes (Lawrence & Cortes, 2014). Thus, decisions on peer-review policies are often based more on the opinions of editors or program chairs, and less on facts, despite their impact on the perceived integrity of the process (Jamieson et al., 2019). Additionally, many authors find the peer-review process inconsistent and somewhat arbitrary (François, 2015; Lawrence & Cortes, 2014). Both conference organizers and the authors who publish in them could benefit from more data on the process.

This article presents data and evidence from statistical observations on the peer-review process for a specific year (2017) and a specific subfield of computing (computer systems or “systems”).^1^ 1Despite its large research output and enormous economic impact, we found no consensus definition for the field of “systems”. For the purposes of this paper, we define it to be the study of computer hardware and software components, which includes research in operating systems, computer architectures, databases, parallel and distributed computing, and computer networks.Like most subfields of computer science (CS), the primary channel for publishing research results in systems is peer-reviewed conferences (Fortnow, 2009; Franceschet, 2010; Vardi, 2009). Many conference policies are similar, such as requiring a minimum of three blind reviews per paper (where the identity of the specific reviewers is hidden from authors). However, conferences can vary considerably in other aspects, such as double-blind reviews, rebuttals, two-phase reviews, etc. These decisions can potentially have dramatic effects on both the quality of the conference and the experience of the authors, but there appear to be conflicting opinions on the effects and tradeoffs of these policies (Mainguy, Motamedi & Mietchen, 2005).

The primary goal of this paper therefore is to analyze the *conference author’s experience*. Its main contribution is an exposition and description of a large-scale author survey of systems researchers. These data could be especially relevant to two groups of people: (1) computer scientists working to better understand the publication process and its effect on their careers and (2) conference chairs wishing to understand the effect of their policies on author experience and diversity.

A secondary goal of this paper is to investigate how the diversity of the respondents affected their survey answers. To this end, we combine our survey data with external data to assess author diversity and potential biases. Specifically, we look at gender, English proficiency, research experience, and geography.

By limiting our scope to conferences in a single subfield, we avoid some variability that might occur across a broader range of disciplines. This important subfield is known for poor gender diversity (DeStefano, 2018; Fox, 2006; Frachtenberg & Kaner, 2021; Mattauch et al., 2020), which gives us a lens by which we can examine any magnified effects of review policy on diversity. Despite this focus on systems, we aimed to analyze a large population to increase the statistical validity and robustness of our measurements. Our complete set includes data from 56 conferences, 2,439 papers, 8,193 authors, and 918 survey respondents.

To the best of our knowledge, this is the first cross-sectional survey of authors across systems conferences. Past studies have concentrated on either wide journal authorship (Editage Insights, 2018; Clarivate Analytics, 2018; Sense about Science, 2019; Solomon, 2014) or a single conference (Beverly & Allman, 2013; Daume, 2015; Papagiannaki, 2007; Parno, Erlingsson & Enck, 2017). We contrast these works with our findings throughout our study.

As an initial exploratory study of the survey, we did not set out to validate specific hypotheses. Nevertheless, there are several research questions for which our data can provide clues and answers across the entire field of systems:

• What are the demographic properties (position, gender, country, English proficiency) of survey respondents?
• Are these demographics, and especially the low number of women, representative of all accepted authors?
• How long does a systems paper take to write? How many attempts does it take to publish?
• How do authors feel about a rebuttal process? What explains differences in opinions?
• How do authors evaluate reviews, and which factors affect these evaluations?
• What are the grade distributions of accepted papers across different categories?
• What are the differences to survey responses for authors of different genders, English proficiency, and publication experience?

### Organization

The next section discusses our methodology and limitations of the survey data. The Results section describes the survey and is organized around the areas of the survey itself. Each subsection lists the survey questions in order, describes the statistics of the responses, and then includes a concise discussion or correlation analysis as applicable. As an initial analysis of the survey, the Discussion section delves into questions of author diversity for which we have data. We believe that the wealth of this dataset leaves more questions unanswered than this expository paper allows, and we discuss some of our future work in the final section. As an additional contribution, most of our data and source code, except for individual survey responses, is available on http://github.com/eitanf/sysconf.

## Materials and Methods

Before issuing our survey, we collected data from various external sources to complement and corroborate its findings, starting with the conferences themselves. We selected 56 conferences from systems and related areas. These peer-reviewed conferences include some of the most prestigious in the field, as well as others for comparison. They vary in scope and size (from 7 to 151 papers), but all are rigorously peer-reviewed and all are from 2017. The complete list of conferences is given in Table 1.

**Table 1:** Conferences in our dataset with their start date, double-blind policy, number of accepted papers, acceptance rate, and survey response rate by papers.

Name	Date	Blind	Papers	Acceptance	Response	Name	Date	Blind	Papers	Acceptance	Response
ASPLOS	2017-04-08	Yes	56	18%	41%	ISC	2017-06-18	Yes	22	33%	45%
ATC	2017-07-12	No	60	22%	22%	ISCA	2017-06-24	Yes	54	17%	31%
CCGrid	2017-05-14	No	72	25%	14%	ISPASS	2017-04-24	Yes	24	30%	38%
CCS	2017-10-31	Yes	151	18%	32%	KDD	2017-08-15	No	64	9%	28%
CIDR	2017-01-08	No	32	41%	41%	MASCOTS	2017-09-20	No	20	24%	25%
CLOUD	2017-06-25	No	29	26%	28%	MICRO	2017-10-16	Yes	61	19%	41%
Cluster	2017-09-05	No	65	30%	20%	Middleware	2017-12-11	Yes	20	26%	35%
CoNEXT	2017-12-13	No	32	19%	31%	MobiCom	2017-10-17	Yes	35	19%	49%
EuroPar	2017-08-30	No	50	28%	34%	NDSS	2017-02-26	Yes	68	16%	54%
EuroSys	2017-04-23	Yes	41	22%	39%	NSDI	2017-03-27	Yes	42	16%	21%
FAST	2017-02-27	Yes	27	23%	52%	OOPSLA	2017-10-25	Yes	66	30%	12%
HCW	2017-05-29	No	7	47%	29%	PACT	2017-09-11	Yes	25	23%	24%
HiPC	2017-12-18	No	41	22%	37%	PLDI	2017-06-18	Yes	47	15%	32%
HotCloud	2017-07-10	No	19	33%	58%	PODC	2017-07-25	No	38	25%	26%
HotI	2017-08-28	No	13	33%	0%	PODS	2017-05-14	No	29	29%	24%
HotOS	2017-05-07	No	29	31%	34%	PPoPP	2017-02-04	Yes	29	22%	48%
HotStorage	2017-07-10	No	21	36%	29%	SC	2017-11-14	Yes	61	19%	41%
HPCA	2017-02-04	No	50	22%	54%	SIGCOMM	2017-08-21	Yes	36	14%	50%
HPCC	2017-12-18	No	77	44%	29%	SIGIR	2017-08-07	No	78	22%	29%
HPDC	2017-06-28	No	19	19%	37%	SIGMETRICS	2017-06-05	Yes	27	13%	30%
ICAC	2017-07-18	No	14	19%	36%	SIGMOD	2017-05-14	Yes	96	20%	31%
ICDM	2017-11-19	Yes	72	9%	26%	SLE	2017-10-23	No	24	42%	4%
ICPE	2017-04-22	No	29	35%	41%	SOCC	2017-09-25	No	45	Unknown	36%
ICPP	2017-08-14	No	60	29%	25%	SOSP	2017-10-29	Yes	39	17%	59%
IGSC	2017-10-23	No	23	Unknown	35%	SP	2017-05-22	Yes	60	14%	38%
IISWC	2017-10-02	Yes	31	37%	45%	SPAA	2017-07-24	No	31	24%	26%
IMC	2017-11-01	No	28	16%	50%	SYSTOR	2017-05-22	No	16	34%	12%
IPDPS	2017-05-29	No	116	23%	28%	VEE	2017-04-09	Yes	18	42%	44%

For each conference we collected data from the Web and program committee (PC) chairs, including review policies, important dates, the composition of its technical PC, and the number of submitted papers. We also collected historical metrics from the Institute of Electrical and Electronics Engineers (IEEE), Association for Computing Machinery (ACM), and Google Scholar (GS) websites, including past citations, age, and total publications, and downloaded all 2,439 papers. From the conference and paper text, we compiled the complete list of authors for all 56 conferences (a total of 8,193 unique authors), as well as their email addresses. These addresses were used not only for the survey’s distribution but also to infer an author’s affiliation, sector, and country of residence. If an email address was not shown in the paper, we attempted to infer the authors’ affiliation from their GS profile when uniquely identifiable. These profiles also provide indirect metrics on the authors’ research experience, such as their H-index (Hirsch, 2005). Finally, we also manually assigned the gender of 97.1% of authors, by looking up their photos and pronouns on the web.^2^ 2We recognize that gender is a complex, nonbinary identity that cannot be captured adequately by just photos or pronouns. However, the focus of this study is on perceived gender, not self-identification, which is often judged by the same simplistic criteria.

We sent our survey to all 5,919 valid email addresses during the summer of 2018, and 918 authors responded. We asked a few demographic questions, as well as questions about their paper and about the review process, repeated for up to three distinct papers from our dataset. Nonresponses to a question were marked as NA.

Of the 809 papers, 161 had responses from multiple authors. Response rates by paper varied considerably among different conferences but appear to be positively correlated with the median number of authors per paper (Pearson’s *r* = 0.37, *p* = 0.0055). In other words, the more coauthors per paper, the more likely it was that at least one author would respond and represent that paper. The distribution of responses per paper was statistically similar to the distribution of coauthors per paper (*t* = 23.54, *p* < 0.001), suggesting that authors were equally likely to respond to the survey, regardless of the paper.

Survey responses from different authors to the same paper were typically identical or very similar, and always tested statistically insignificant in aggregate. In five papers, the responses from different authors were so inconsistent across questions that we elided them from our data. These inconsistencies relate mostly to the paper’s history, whereas responses to most other questions remain consistent across respondents.

### Limitations

Our methodology involves several limitations and tradeoffs worthy of mention. First, by focusing only on systems, we may be limiting the applicability of our findings to this subfield. By focusing on a single year, we cannot report trends. These choices were deliberate, to eliminate extraneous variability in our data. Second, our survey is subject to selection bias (representing only authors who responded to the survey or to each question). Since we found no statistically significant demographic differences between survey respondents and the group of all authors, we believe the effect of this bias is minimal (see also Daume, 2015; Papagiannaki, 2007). Third, the effort involved in compiling all of the data in preparation for the survey took nearly a year, by which time some authors reported difficulty recalling some details, leading to fewer responses. Fourth, the manual assignment of genders is a laborious process, prone to human error. However, automated approaches based on first names and country can have even higher error rates and uncertainty, especially for female and Asian names (Huang et al., 2019; Karimi et al., 2016; Mattauch et al., 2020). In fact, for the 786 respondents who provided a binary gender, we found no disagreements with our manual gender assignments.

Last, but certainly not least, is survivorship bias. Since we only polled authors of accepted papers, we have no information on all submitted papers. Our survey data is insufficient to distinguish between the demographics of accepted and rejected authors, which leaves the door open to undetected biases in the peer-review process. That said, we found no difference in the demographics of accepted papers between otherwise similar conferences with double-blind or single-blind review policies. This indirect evidence reduces the likelihood that the demographic section of the survey would be answered differently for rejected papers. Other survey sections on paper history and review process may prove more sensitive to survivorship bias. We therefore limit any conclusions we draw in this study to accepted authors only. Even with this restriction, it can be instructive to compare the responses across different demographics within accepted authors.

We found very few controlled studies that evaluate the peer-review process on both accepted and rejected papers, and they are typically limited in scope to one conference or journal (Parno, Erlingsson & Enck, 2017; Tomkins, Zhang & Heavlin, 2017). We chose an observational approach instead, which lets us examine an entire field of study, but at the cost of survivorship bias and experimental control. We believe both approaches to be complementary and valuable.

### Ethics statement

This study and the survey questions were approved by the Reed College IRB (number 2018-S13). As an opt-in email survey, participants could choose to share their responses with us after they were informed of the questions and the purpose of the survey. All of the individual responses have been anonymized. The data that is shared in the supplementary material was collated and collected from publicly available sources on the Web.

## Author Survey Results

### Demographic questions

We asked three demographic questions to evaluate their role in the review experience. We intentionally kept these questions to a minimum to reduce the risk of priming or selection bias.

#### Which best describes your position during 2017?

As shown in Table 2, about one-third (36.2%) of the respondents were students in 2017, another third or so were professors of various ranks (34%), and the rest were distributed between all other categories, including unknown. For comparison, we looked at the inferred affiliation of 7,026 total authors with an identifiable email affiliation. Of these, 13.9% had an industry affiliation, compared to 13.6% of the non-NA survey respondents (*χ*^2^ = 0.059, *p* = 0.807). The difference for government researchers is a little larger: 4.8% by affiliation vs. 6.4% among survey respondents, but still not significant enough to suggest selection bias by position (*χ*^2^ = 2.977, *p* = 0.0845).

**Table 2:** Distribution of respondent positions.

Response	Count	Ratio
Government Researcher	41	4.5%
Industry Researcher	115	12.7%
Professor	114	12.6%
Associate Professor	71	7.8%
Assistant Professor	124	13.7%
Postdoctoral Researcher	35	3.9%
Student	329	36.2%
Other	19	2.1%
NA	60	6.6%

Systems is a field with numerous practical applications and commercial implications. It is not surprising therefore to find a large proportion of researchers in industrial and government positions, contributing to author diversity across sectors.

#### What is your gender?

Among those who provided a binary response, 10.5% chose “Female” (Table 3). In our manually assigned gender data of all of the authors, 11% were female. These two proportions are not statistically different (*χ*^2^ = 0.156, *p* = 0.693), leading us to believe that significant selection bias by gender is unlikely.

**Table 3:** Respondents’ gender.

Response	F	M	Other	NA
Count	83	703	4	112
Ratio	9.2%	77.9%	0.4%	12.4%

#### What is your English level proficiency?

Of the 848 non-NA respondents, 31% of respondents chose “Native” for their English level proficiency. There appears to be no gender or position difference in the response to this question.

We also asked (and checked) for each paper whether there was any native English speaker among its coauthors. From this question, we estimate that approximately 54% of papers had at least one native-speaking author.

### Paper history

#### How many months did it take to research and write?

The responses to this question (Table 4) exhibited more variance among different coauthors of the same paper than any other question, although typically by no more than 3 months. The response to this question was not significantly associated with the team size (number of coauthors) or lead author’s experience, gender, or sector.

**Table 4:** Months of research.

Response	1–3	4–6	7–9	10–12	13+	NA
Count	42	162	119	126	164	191
Ratio	5.2%	20.1%	14.8%	15.7%	20.4%	23.8%

#### How many conferences/journals was it submitted to prior to this publication?

It is instructive to see that at least 40% of papers with responses had been rejected at least once (Solomon, 2014; Wallach, 2011), with one respondent taking as many as 12 attempts to reach publication (Table 5). We also observed a tendency of papers with a longer submission history of having a longer research history (previous question), perhaps conflating the two variables in respondents’ mind.

**Table 5:** Number of paper’s prior submissions.

Response	0	1	2	3	4	5+	NA
Count	353	153	65	27	8	7	191
Ratio	43.9%	19%	8.1%	3.4%	1%	0.9%	23.8%

#### Please type in their names [of the rejecting conferences]

Because of the unstructured responses to this question, quantitative analysis is challenging. As an example, we focus our attention on the area of computer architecture alone. Four of the leading conferences are represented in our dataset and are of similar size and acceptance rates. We note that most papers that had been previously rejected from these conferences, had been mostly submitted to one of these four as well.

**Figure 1:**
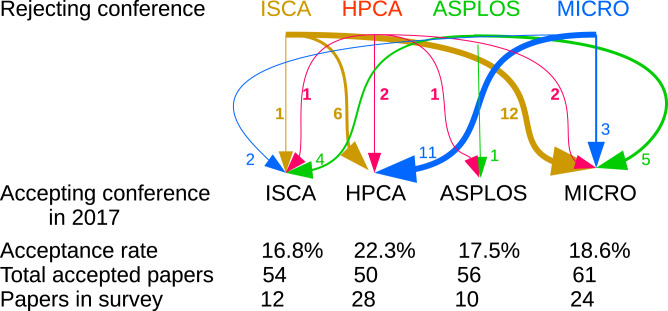
Prior submission counts for architecture conferences. Arrows show in relative thickness and attached number how many papers that had been rejected in the top row’s conference were accepted in the bottom row’s conference. For example, 6 papers that had been rejected from ISCA were accepted to HPCA in 2017, out of the 28 HPCA’17 papers for which we have responses.

As Fig. 1 shows, these relationships work both ways, meaning that many papers were accepted after previously being rejected from equivalent (or even the very same) conferences. This fact can be interpreted both positively and negatively. Some respondents expressed frustration that the peer-review process can appear arbitrary (Anderson, 2008; François, 2015; Gans & Shepherd, 1994; Lawrence & Cortes, 2014; Vardi, 2009; Vines, 2011). Other authors opined that effective peer review provides feedback that improves the paper for the next submission. Most of the papers had been rejected at least once prior to their acceptance in 2017, which perhaps helps to explain why authors’ views on the process were mixed. This fact could also support an argument that selection bias in this survey played a lesser role in painting authors’ reported experience one way or another, because even though these are all accepted authors, most of them experienced the rejection of the subject paper as well.

### Rebuttal process

#### Did the conference allow you to address reviewers concerns before final acceptance notice?

Of the 710 non-NA responses, 57.3% chose “Yes.” Contrast this with the conferences, of which only 18 offered a formal rebuttal option (33.9% when weighted by papers). The discrepancy may be explained by some authors who specifically explained answering “Yes” to this question despite the lack of a formal rebuttal policy, because the conference had a “provisional acceptance” policy or mandatory revisions guided by a PC “shepherd.” Although this response type is clearly different than a formal rebuttal, limiting our analysis to only formal rebuttals does not meaningfully change our results.

Approximately 96.45% of the “Yes” respondents also reported that they took advantage of the rebuttal option. The few who did not take advantage received higher overall acceptance score on average, (83.3% vs. 66.2%, *t* =  − 1.6, *p* = 0.18), possibly obviating the need to rebut (Daume, 2015).

There were no statistically significant differences in responses to this question by position, English proficiency, or gender, although only men chose not to rebut (16 authors, *χ*^2^ = 0.911, *p* = 0.34). These 16 men appear slightly less experienced than their peers, with a median H-index of 8, compared to 10 for all authors, (*t* =  − 1.408, *p* = 0.184) and are mostly academics (12 authors). However, the group is probably too small to characterize it conclusively.

#### Did you find the response process helpful?

Of the non-NA responses, 89.7% were affirmative. This high percentage may be a little surprising, considering how many PC chairs and authors alike commented privately on how little difference rebuttals make (Daume, 2015; Shah et al., 2018). One cautionary reminder is that the survey and statistics exclude rejected papers, which could lead to survivorship bias. It is quite plausible that authors of rejected papers were less enthused about the rebuttal process. However, even among authors of accepted papers there are some noteworthy differences between those who found rebuttals valuable and those who did not.

Professors comprise only 35% of the respondents who found rebuttals helpful, compared to 53% among those who did not (*χ*^2^ = 5.44, *p* = 0.02). In contradistinction, students found rebuttals more helpful (42% vs. 17%, *χ*^2^ = 10.36, *p* = 0.0013), perhaps because of their lack of experience. Junior researchers possibly also feel more pressure to bring their paper to publication than tenured and senior researchers.

More generally, the experience level of authors who found rebuttals helpful, as measured by median publications count in their GS profile, is about half that of those who did not (23 vs. 43, *t* = 1.55, *p* = 0.13). We have also collected information on which authors serve on PCs in any of our conferences, as another measure of experience. This information agrees with the previous metric. Authors satisfied with the rebuttal process serve on an average of 0.3 PCs, compared to 0.6 PCs for authors who were not (*t* = 1.89, *p* = 0.066), which is consistent with the mixed opinions we got directly from PC chairs on the question of rebuttals.

Nonnative English speakers were also more likely to find the rebuttals helpful (92% vs. 85%, *χ*^2^ = 4.741, *p* = 0.0294), perhaps because it allowed them to address gaps in communication. This difference also extends weakly to the entire team: 91% of responses where no team member was a native English speaker found the rebuttal helpful, vs. 89% in responses from the other teams.

Rebuttal helpfulness does appear to be related to the conference. When limiting ourselves to the eleven conferences that had a formal rebuttal process and at least ten unique authors responding to this question, three conferences had higher-than-average dissatisfaction rate with the rebuttal process: ASPLOS, ISC, and SOSP. Conversely, in four conferences, no more than 8% of respondents were dissatisfied with the rebuttals: MICRO, PPoPP, SC, and PLDI.

When asked to explain their previous answer, the respondents varied. The main themes that emerged from the positive responses were that rebuttals allowed for clarifications, increased review scores, and improved the communication of specific points in the paper. One PC chair also thought rebuttals elicit better initial reviews and better PC discussion. The main negative themes were that rebuttals rarely change reviewers’ minds and that the process was still opaque and arbitrary.

### Review quality assessment

The following questions, one per review and paper, were designed to assess the quality of the reviews.

#### How many reviews did this paper receive?

The papers in our dataset average more than four reviews per paper (Table 6), far better than the typical 2+ reviews in an average CS journal (Clarivate Analytics, 2018, p. 21). This could partially explain the attractiveness of conferences over journals, at least in systems. Authors were also asked to qualitatively approximate *how long each review* was (Table 7). It is encouraging to find over half of the non-NA responses showing one or more pages per review, whereas only approximately 15.9% of reviews were reported to be less than half a page.

**Table 6:** Number of reviews received per paper.

Response	0	1	2	3	4	5	6+	NA
Count	2	1	5	206	204	167	65	154
Ratio	0.2%	0.1%	0.6%	25.6%	25.4%	20.8%	8.1%	19.2%

**Table 7:** Distribution of review lengths.

Response	Count	Ratio
1-2 Paragraphs	51	6.3%
Half a Page	118	14.7%
A Page	181	22.5%
Multiple Pages	65	8.1%
NA	389	48.4%

#### How well did the reviewer understand the paper, in your estimation?

Of the minority of reviews that missed major points or worse (Table 8), 59.7% were short, spanning half a page or less. This correlation demonstrates the relationship between review quality and length (*χ*^2^ = 55.325, *p* < 0.0001) (Hames, 2008; Papagiannaki, 2007). Still, longer is not always better or necessary, as these short reviews still comprise 40.9% of the “perfect understanding” reviews, whereas multipage reviews only comprise 15.3%.

**Table 8:** Reviewer understanding.

Response	Count	Ratio
Perfectly	570	30.1%
Missed some minor points	684	36.1%
Misunderstood major points	105	5.5%
Probably didn’t read it	14	0.7%
NA	520	27.5%

As for paper history, the better-understood papers appear to have had a longer history in terms of prior submissions (*t* = 2.49, *p* = 0.014), as well as in terms of months researched. Conceivably, previous rejections have helped improve the communication of a resubmitted paper.

#### How helpful did you find this review for improving the paper?

Table 9 shows that accepted authors found most of their reviews at least somewhat helpful. The helpfulness of a review is closely linked to its reported level of understanding (*χ*^2^ = 284.53, *p* < 0.001), which in turn also implies that it is closely linked to the review’s length (*χ*^2^ = 267, *p* < 0.001). This result is consistent with other surveys of journal authors (Editage Insights, 2018; Sense about Science, 2019).

**Table 9:** Review helpfulness.

Response	Count	Ratio
Very helpful	465	34.1%
Somewhat helpful	718	52.7%
Not at all	180	13.2%

#### How fair would you say the review was?

Fairness in reviews is a high priority for the systems community (Jerger et al., 2017), and most of our respondents thought their reviews were fair (Table 10). Once more, the perception of a review’s fairness is closely tied to that of the reviewer’s understanding (*χ*^2^ = 766.81, *p* < 0.001) and helpfulness (*χ*^2^ = 281.64, *p* < 0.001).

**Table 10:** Review fairness.

Response	Count	Ratio
Fair	1019	75.4%
Somewhat fair	280	20.7%
Unfair	49	3.6%
Very unfair	3	0.2%

Only 52 of non-NA responses (3.85%) ranked a review as ‘Unfair’ or ‘Very unfair.’ However, this relatively low number may be distorted by survivorship bias more than for any other question in this survey. Of these responses, SOSP stands out as the conference with most ‘Unfair’ reviews (5, or 7.58%) and ICPE as the conference with the highest percentage (2, or 28.57%). One other notable aspect of these negative responses is that only one came from a woman (1.9%).

### Review scores

We asked respondents to upload their reviews’ text or to fill in the actual scores that they received in the reviews of up to six reviews per paper and in seven different categories, when applicable. Not all of the conferences require all categories in their review forms, and the conferences do not all use consistent wording, so we chose whichever one of the seven categories appeared closest in meaning to the conference’s form. These categories generally stand for the following:

1. Overall score or acceptance recommendation (often ranging from “strong reject” to “strong accept”).
2. Technical merit or validity of the work.
3. Presentation quality, writing effectiveness, and clarity.
4. Foreseen impact of the work and potential to be of high influence.
5. Originality of the work, or conversely, lack of incremental advance.
6. Relevance of the paper to the conference’s scope.
7. Confidence of the reviewer in the review.

**Figure 2:**
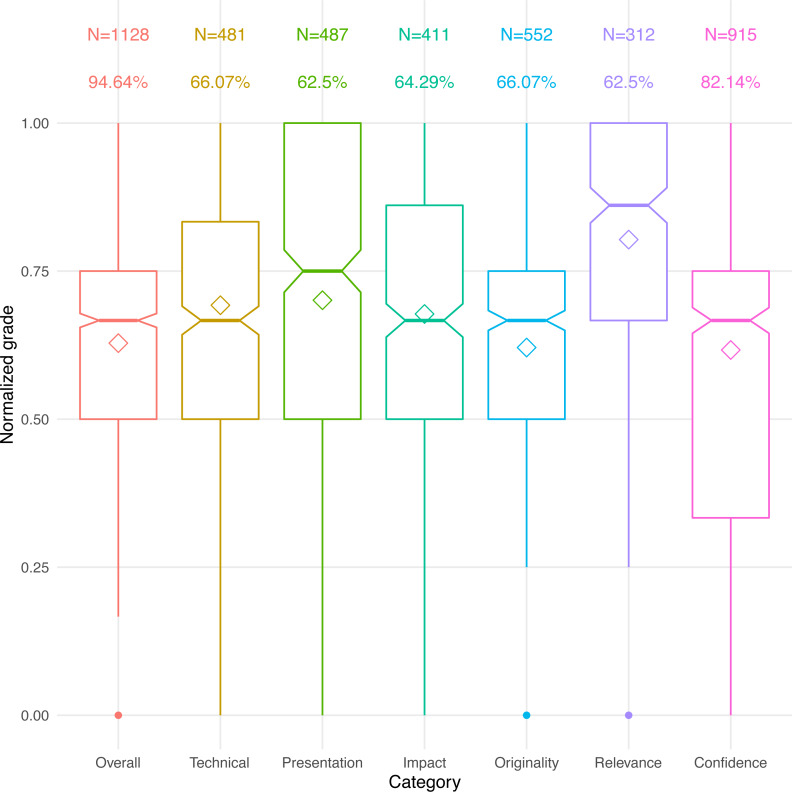
Normalized scores and response distribution. Diamonds represent mean scores. Bars represent median scores, with a notched 95-pct confidence. N is the number of scores received in each category. Shown below N is the percentage of conferences that used each grade category.

All scores were normalized so that the lowest grade in a category always received 0 and the highest always 1. The distributions of these normalized scores are depicted in Fig. 2. Keep in mind, however, that the transcription of reviews, scaling, and calibration process were error-prone, possibly introducing some noise to these responses.

Not surprisingly, all of the papers average above 50% for all of the scores—after all, the papers have all been accepted (Langford, 2012; Vines, 2011). The interquartile range for the overall grade is 0.5–0.75, meaning that half of the papers probably got accepted with an overall recommendation somewhere between “weak accept” and “accept.” Perhaps more surprisingly, approximately 10% of the papers were accepted despite a low (<0.5 average) acceptance recommendation, and approximately 21% of the accepted papers had low reviewer confidence (<0.5 average). However, the confidence ranking may be related to the seniority of the reviewer rather than the quality of the paper itself, leading to wider variance (Shah et al., 2018).

It is illuminating to see that there is no correlation between a paper’s overall grade and the number of past rejections (*r* =  − 0.07, *p* = 0.016). If multiple submissions do indeed improve a paper’s quality, as we suggested in the understanding question, they appear to only bring it to the same level of evaluation as other accepted papers in the same conference. Once the paper is accepted, the improvement process is presumably halted.

Another observation is that the “relevance” grade may be mostly irrelevant, both because of its narrow distribution, and because of the low number of conferences that ask for it. Conceivably, an out-of-scope paper could simply get rejected and excluded from our dataset. Alternatively, this grade could be so important that papers are at a much higher risk of rejection if they are mismatched with the conference’s scope, even if they rank well in the other categories. Unfortunately, without data on rejected papers we do not have enough information to discriminate between these two extremes.

## Discussion and Author Diversity

In this section we address differences in survey responses based on aspects of author diversity that arise from the available data.

### Gender

Women represent only approximately 20–30% of CS researchers overall (Wang et al., 2019). In our data, the percentage is about half that, with only 10.5% female survey respondents. What factors could explain this lower ratio?

One potential explanation is selection bias: women might be less inclined to respond to this survey. However, the percentage of women across respondents and nonrespondents alike, 11%, is actually very close.

Another explanation may be that women publish less than men in systems. Indeed, women in our dataset did average fewer total past publications: 0.85 compared to men’s 1.06. Nevertheless, this gap is not large enough to explain the 2–3x representation gap with the rest of CS and is not unique to systems (Elsevier, 2017).

A third explanation could be that female authors’ papers are rejected at a higher rate than males’. We cannot test this hypothesis directly without data on rejected papers. However, three pieces of evidence weaken this explanation:

1. The ratio of women in the 25 double-blind conferences, where reviewers presumably remain oblivious of the authors’ gender, is in fact slightly lower than for single-blind conferences (10.06% vs. 10.94%, *χ*^2^ = 3.032, *p* = 0.22). This ratio does not support an explanation that reviewers reject females at a higher rate when they can look up the author’s gender.
2. When we limit our observation to lead authors only, where the author’s gender may be more visible to the reviewers, the ratio of women is actually slightly higher than in the overall author population (11.25% vs. 10.48%, *χ*^2^ = 1.143, *p* = 0.285). If we assume no differences in the submission rates to a conference based on gender, then female lead authors appear to suffer no more rejections than male authors.
3. We found no statistically significant differences in the overall acceptance grades of women and men (*t* = 0.291, *p* = 0.772), even when limiting to lead authors (*t* = 0.577, *p* = 0.566), papers accepted on their first attempt (*t* = 0.081, *p* = 0.935), or single-blind reviews (*t* = 1.159, *p* = 0.253). This equitability extends to most other grade categories, except for originality (*t* = 4.844, *p* < 0.0001) and technical merit in single-blind conferences (*t* = 2.288, *p* = 0.0294). In both categories, women scored significantly higher than men. It remains unclear whether there is any causal relationship here, and if so, in which direction; do women have to score higher than men in the technical categories to be accepted in single-blind conferences, or do women submit higher-quality papers to begin with? At any rate, this small difference is unlikely to explain the 2–3x difference in women’s ratio compared to CS, but it does provide a case for wider adoption of double-blind reviewing.

These distinctions were not the only gender differences in our survey. Women also reported reviewers as somewhat more understanding, helpful, and fair than men did (*χ*^2^ = 12.01, *p* = 0.062, *χ*^2^ = 10.87, *p* = 0.028, and *χ*^2^ = 7.06, *p* = 0.32, respectively). On the other hand, papers authored by women averaged a few more prior submissions: 0.95 compared to men’s 0.64 (*t* = 3.34, *p* = 0.001). Note, however, that review quality and prior submissions are strongly linked. In other words, a paper with a longer submission history tends to rate higher on reviewer understanding, helpfulness, and fairness. When correcting for submission history length, these gender differences lose statistical significance.

In summary, our data does not exhibit large statistical gender differences in the review process, and in particular it does not help to explain the large gender gap in systems. Addressing this problem may require focusing our attention elsewhere (Ceci & Williams, 2011).

### English proficiency

Another aspect of diversity in scientific publication is English-level proficiency (Lee et al., 2013; Murray et al., 2019). All of the papers, reviews, and communications in our conferences were conducted in English, but many authors and reviewers are nonnative English speakers (NNES). The effective use of language can affect both reviewers’ understanding of the works and authors’ understanding of the reviews (Crovella, 2008; Editage Insights, 2018; Flowerdew, 1999 Flowerdew, 2001). How does the author experience vary based on this factor?

At least in our dataset, the answer appears to be “not much.” From an objective grading perspective, all but one of the review categories exhibit very similar distributions, both for teams with native English speakers and for teams with none. These categories include the presentation grade (*t* = 0.638, *p* = 0.524), where language skills presumably would make the most difference. The only exception was the originality grade, where teams with no native speakers averaged a normalized grade that was slightly higher than the native speakers’ teams (0.65 vs. 0.596, *t* = 2.578, *p* = 0.0102).

As for the subjective experience of authors, NNES do feel differently about how well reviewers understand their work (*χ*^2^ = 9.984, *p* = 0.0187), but perhaps not in the way that we would expect; of those reviews with reportedly poor understanding, only 2.4% were from all-NNES teams, compared to 35.1% all-NNES teams in the better-understood reviews. The overall rate of NNES teams among survey responses was 45.8%, so clearly most of them did not feel misunderstood. Similar to women, NNES average higher prior submissions, 0.71, compared to native speakers’ 0.65 (*t* = 1.21, *p* = 0.23), which may be the stronger explanatory variable.

We also tried to look in the opposite direction: how does the English level of the reviewers affect how well understood the authors feel? We do not know who reviewed whose paper, or even a reviewer’s native language or nationality. However, we can try to estimate it indirectly by looking at their affiliation’s country. We first guess the country of residence of reviewers by looking at their email affiliation, extract a country when possible, and look up whether this country includes English as one of its official languages. We then look at the conference PC overall demographics and assign each conference a value corresponding to the percent of PC members affiliated with an English-speaking country. Program committees range from 91% English speakers (SOCC) to 24% (EuroPar), and average 68.5%. As it turns out, this proportion has no significant association with the reported understanding level of the reviews for the conference.

These negative findings could suggest that in the overall picture of systems research, English proficiency is merely one resource in the multidimensional skill set required to publish successfully (Bardi, 2015; Ferguson, Pérez-Llantada & Plo, 2011; Rozycki & Johnson, 2013) and that the binary distinction of native/nonnative speaker may be inadequate to capture even this skill alone.

### Publication experience

**Figure 3:**
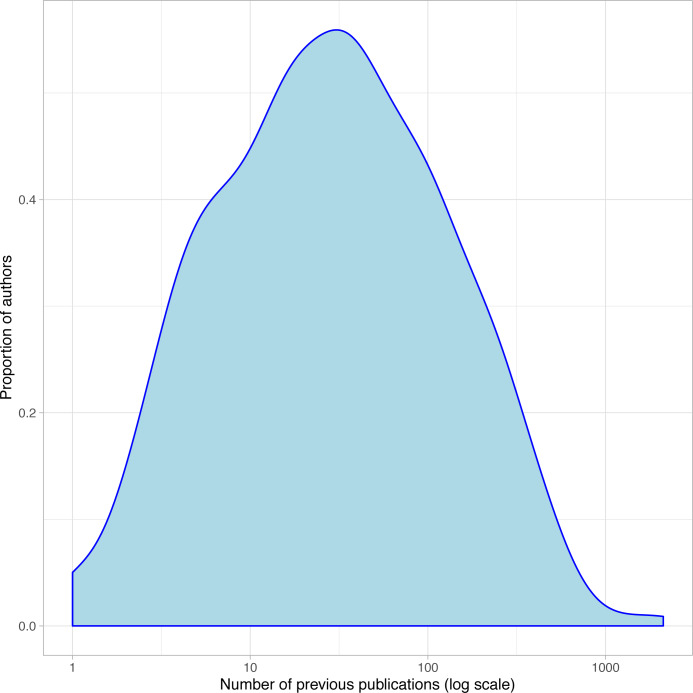
Distribution of past publications of all authors, near the time of their first 2017 publication.

As mentioned in the Methods section, we collected data from authors’ GS profile whenever available and uniquely identifiable (66.4% of our survey respondents). We can use this bibliometric data as an approximate proxy for the previous research experience of authors. For example, Fig. 3 depicts the distribution of one such metric, the number of previous publications of each author (circa their conference’s date), which appears approximately log-normal.

    Since we collected this metric for all authors, not just survey respondents, we can compare the distributions for both populations. Both distributions are similar enough to lead us to believe that no selection bias by experience occurred in this survey (*t* = 2.2, *p* = 0.028).

We can also look at the more complex H-index metric (Hirsch, 2005) to evaluate differences in response rate by researcher seniority. Some 35.7% of respondents had an H-index of 5 or less, roughly corresponding to the percentage of self-identified students. This percentage is nearly identical in the overall author population (33.4%), again confirming that the large number of students in our survey is representative of the author population.

This large representation of students is important in light of our previous findings about the differences between survey responses of students and of more experienced researchers. For example, students in our survey overwhelmingly prefer a rebuttal process. More experienced researchers commented in the survey that they tend to value this process less, which may affect conference policies, because those are also decided by experienced researchers. Nevertheless, their high value to inexperienced researchers (as well as NNES) may render the effort worthwhile (Langford, 2013).

As previously discussed, we found no correlation between the experience of a paper’s lead author and its research or submission history in months and submissions. The same is true when comparing the number of past rejections with the past publications of a paper’s most-experienced author (*r* = 0.004, *p* = 0.91), least-experienced, mean and median experience. We also found no correlation between an author’s experience and their response to the understanding or helpfulness of the reviews. We believe that these negative findings are an overall positive indication that the peer-review process is fair and blind to experience, although a full analysis requires incorporating rejected papers as well.

We did find a weak association, however, between authors’ experience and the reviews’ perceived fairness (*χ*^2^ = 14.662, *p* = 0.0231), which was also observed in the ISCA community for fairness and helpfulness (Jerger et al., 2017).

### Geographical regions

Although we did not specifically ask authors for their country of residence, we can infer this information for most authors from their email addresses. We can then aggregate authors based on the region of the world that their email affiliation belongs to and compare the distribution of ratios between survey respondents and all of the authors. Table 11 shows these distributions (omitting any authors with unidentifiable country and any regions with two authors or fewer).

**Table 11:** Number and percentage of survey respondents and total authors by geographical region, in descending number of total authors.

Region	Respondents	Percentage	All authors	Percentage
Northern America	471	57.7	3510	56.0
Eastern Asia	80	9.8	831	13.2
Western Europe	103	12.6	826	13.2
Northern Europe	55	6.7	363	5.8
Southern Europe	42	5.1	235	3.7
Western Asia	14	1.7	128	2.0
Southern Asia	18	2.2	102	1.6
South-Eastern Asia	7	0.9	89	1.4
Australia and New Zealand	7	0.9	85	1.4
South America	9	1.1	64	1.0
Eastern Europe	10	1.2	40	0.6

It is encouraging to see that the two distributions are fairly similar (*t* =  − 1.603, *p* = 0.139), which suggests that any selection bias based on geographical region is also limited.

Unsurprisingly, most of these researchers hail from the West, much more so than in other fields (Clarivate Analytics, 2018). One possible explanation is that systems research can require expensive hardware, and is therefore more likely to occur in the well-endowed research institutions and companies of the developed world. Regardless of explanation, this data shows a strong dissonance between country population and representation in published systems research, leading in turn to poor geographical diversity.

A final point of interest is to combine all these metrics to look at NNES who migrate to or reside in an English-speaking country. Of the 713 respondents with a identifiable email affiliation, 439 reside in the US, and 51 more in the UK, Canada, and Australia. Of the US-based researchers, 59.7% identify as NNES. This group of migrants and visitors exhibits different demographic characteristics than the native US researchers. These migrants show a higher rate of students (55% vs. 29.9%, *χ*^2^ = 25.727, *p* < 0.0001), which coincides with a lower research experience (median H-index of 6 vs. 11, *t* =  − 4.606, *p* < 0.0001), and somewhat higher rate of academic sector affiliation (92.4% vs. 87.6%, *χ*^2^ = 2.281, *p* = 0.131). These immigrants and visitors, however, exhibit the same gender imbalance as the locals, with a female respondent rate of 13% vs. 12.4%, *χ*^2^ = 0.001, *p* = 0.982).

## Conclusions and Future Work

This paper presented a new survey of conference authors, exposing the experience of authors across a large section of computer systems. We placed a strong emphasis on examining responses across various author demographics and found no selection bias based on the authors’ gender, experience, position, geographical region, or paper. We think these responses are representative of authors of accepted papers throughout the field of systems, and can be used to inform future conference policies, such as double-blind reviews and author rebuttal. The former remains an important research question, and we plan to explore it with our survey data in future work.

Most survey takers found the opportunity to respond to reviewers valuable, even if it did not change their review grades. The implication for PC chairs, and by extension, educators, may be that while a response process to technical feedback is of little value to experienced practitioners, novices do find it overwhelmingly helpful. Students are well represented in this survey, possibly because systems research often requires elaborate implementation efforts, including multiple graduate students. Students’ responses to the survey could be useful for conferences with an educational mission to better address this target audience. A related finding is that longer feedback is generally perceived as more helpful, understanding, and fair, which in turn may serve as another factor in improving students’ experience.

Overall, we found that published authors in systems exhibit a good mix of work sectors, research experience, and English proficiency, but poor diversity across gender and geographical regions. Women in particular represent an alarmingly small group of authors in systems research, and this paper looked at whether the peer-review process plays a role in this underrepresentation, as has been found in some grant and job evaluations (Lee et al., 2013). For female authors of accepted papers, we found that their papers tend to have a slightly longer submission history. However, we found little evidence of negative outcomes in the reviews that they received or experience they perceived, even when their identity is known to the reviewers.

Nonnative English speakers also appear to experience no specific adverse effects from peer review, and in fact often report more positively on their experiences than native speakers. Both of these findings can help focus the diversity effort on other policies, at least for accepted authors. The larger question of nativism in peer review requires data on rejected papers, and is not answered in this paper.

In terms of conference policies, the two main qualitative conclusions that we draw from the quantitative results are that from the authors’ perspective, review response or rebuttal can be very valuable, and that short reviews often are not. Conference chairs may take these findings into consideration in their review policies, especially if they intend to attract junior researchers.

This dataset remains rich for exploration of the many questions that fell outside the scope of this paper, such as the following:

• Why is the representation of women in systems so low?
• Do women actually *need* to receive higher technical scores in their reviews just to be accepted to single-blind conferences?
• What are the effects of double-blind reviewing on the quality of reviews, conferences, and papers?
• What other publication differences and commonalities exist between systems and the rest of CS?
• How do review grades correlate across categories?
• How might reviewer load affect our results?
• How do any of these factors affect the eventual success of a paper, as measured by awards or citations?

We plan to address these questions and others in subsequent research. Our hope is that by opening up all of the nonprivate data we collected, we also open the door for other researchers to validate our results, extend them, or collaborate on future studies.

##  Supplemental Information

10.7717/peerj-cs.299/supp-1 Supplemental Information 1 Full text of survey questionnaire

10.7717/peerj-cs.299/supp-2 Supplemental Information 2 Data and source code for project This is a snapshot of the github repository that includes the data and source code required to reproduce this paper (except for confidential survey data). The snapshot represents commit 6663a253f1ac4dc351a78ccc74c0de80c7cc06ad of http://github.com/eitanf/sysconf. The most pertinent article files are under pubs/diversity-survey/.

10.7717/peerj-cs.299/supp-3 Supplemental Information 3 Anonymized survey data The legend for all fields is in File S4.

10.7717/peerj-cs.299/supp-4 Supplemental Information 4 Metadata description of the fields in survey responses file

## Additional Information and Declarations

### Data Availability

The following information was supplied regarding data availability: All the code and data (except confidential survey responses) are available at Github: http://github.com/eitanf/sysconf. A snapshot of this repository is also available as a Supplementary File. Additionally, the complete survey questionnaire and anonymized individual survey responses are available as a Supplemental File.
