# Supplementary material for: A survey of accepted authors in computer systems conferences
Source: PeerJ Comput Sci. 2020 Sep 28;6:e299. doi: 10.7717/peerj-cs.299 (PMC7924675; doi:10.7717/peerj-cs.299)
Supplement: Supplemental Information 1 [file peerj-cs-06-299-s001.pdf]

Author Data - Full Release

This survey will ask questions related to the peer-review process for the papers you have authored in our data set. Your responses are extremely valuable to identify and understand variations in the peer-review process among different conferences, and their effects on the author's experience. The survey should take only a few minutes to complete. You can also close your browser window at any point to quit, or reload this link to resume where you left off.

Confidentiality
All individual survey responses are confidential, and identifying information will never be shared with any other party. We plan to publish the survey results, but with no identifying information about the respondents. If you have questions about the study, feel free to contact sysconf-survey@reed.edu.
This study has been approved by Reed College's Institutional Review Board (IRB) on April 10th, 2018.

Tell us a little about yourself

Which best describes your position during 2017?

- ○ Student
- ○ Assistant Professor
- ○ Associate Professor
- ○ Professor
- ○ Industry Researcher
- ○ Government Researcher
- ○ Other ____

[If choice other] Please expand on other:

____

What is your English level proficiency?

○ Native

○ Non-native

What is your gender?

○ Male

○ Female

○ Other

○ Prefer not to answer

Next, we'd like to ask you about your experience as an authors of these papers:
[Papers published in 2017]

[For each published paper, the following questions were asked]

Please answer questions below related to the paper "*[Paper]*" published in *[Conference]*'17:

Paper History

About how many months did it take to research and write this paper?

- ○ 1-3
- ○ 4-6
- ○ 7-9
- ○ 10-12
- ○ >12

How many conferences/journals was it submitted to prior to this publication?

- ○ 0
- ○ 1
- ○ 2
- ○ 3
- ○ 4
- ○ 5
- ○ 6
- ○ 7
- ○ 8
- ○ 9+

Please type in their names.

____

[If “non-native” speaker option chosen]

Was one of your co-authors a native English speaker?

- ◯ Yes
- ◯ No

How many reviews did this paper receive?

- ◯ 0
- ◯ 1
- ◯ 2
- ◯ 3
- ◯ 4
- ◯ 5
- ◯ 6+

Page Break

Rebuttal

Please answer the questions below related to the rebuttal given by *[Conference]*'17

[This section shows only once per conference, if it has been submitted already for the same conference then it will not appear]

Did [*Conference]*'17 allow you to address reviewers concerns before final acceptance notice?

○ Yes

○ No

Did you take advantage of the opportunity to respond to reviews?

○ Yes

○ No

Did you find the response process helpful?

○ Yes

○ No

[If response "Yes"]

Why was it helpful?

____

____

____

[If response “No”]

Why was it not helpful?

Review Quality

Please locate your reviews and answer the following questions about each review:

[For each review the following questions were asked]

Reviewer *#[n]*

About how long was the review? (assume a page is about 500 words)

- ○ Multiple Pages
- ○ A Page
- ○ Half a Page
- ○ 1-2 Paragraphs

How well did the reviewer understand your paper, in your estimation?

- ○ Perfectly
- ○ Missed some minor points
- ○ Misunderstood major points
- ○ Probably didn’t read it

How helpful did you find this review for improving the paper?

- ○ Very Helpful
- ○ Somewhat Helpful
- ○ Not at all

How fair would you say the review was?

- ○ Fair
- ○ Somewhat Fair
- ○ Unfair

○ Very Unfair

Review Grades

We are planning to aggregate reviewer quantitative evaluations across papers and conferences to correlate them with different conference policies. You are not required to provide us the reviewer evaluations, but it would be extremely valuable if you did. Please paste your review in the text box and we will process it or upload it below.

____

____

____

____

____

[If no text – ask for upload]

Press the button below to upload your review. Please only upload a text or pdf file.

[If no upload – ask for manual input]

Fill out the grades for the categories that best match the first review. For example, if the grade for presentation/readability had four categories: "Poor", "Acceptable", "Good", "Excellent" and the paper received "Excellent", then it would be classified as 4 out of 4. Alternatively, you can hit the back arrow to upload your reviews and we'll process the grades for you.

	Grade	Out Of
Overall Acceptance Recommendation/Grade		
Technical soundness		
Presentation/Readability		
Impact/Significance/Timeliness		
Originality/Novelty		
Relevance to conference's scope		
Reviewer Confidence		

Please add any additional insights about the review process for this specific paper, such as your opinion on how fair and accurate the reviewers were in grading your paper.

____

____

____

____

____

<u>Survey Complete!</u>

If you have additional comments or questions about the review process or this study, please type them here.

____

____

____

____

____

Would you like to receive an email at *[Email]* when the results of the survey are available?

○ Yes

○ No

Would you like to be considered for the gift card drawing?

○ Yes

○ No
