# Supplementary material for: A survey of accepted authors in computer systems conferences
Source: PeerJ Comput Sci. 2020 Sep 28;6:e299. doi: 10.7717/peerj-cs.299 (PMC7924675; doi:10.7717/peerj-cs.299)
Supplement: Supplemental Information 2 — This is a snapshot of the github repository that includes the data and source code required to reproduce this paper (except for confidential survey data). The snapshot represents commit 6663a253f1ac4dc351a78ccc74c0de80c7cc06ad of http://github.com/eitanf/sysconf. The most pertinent article files are under pubs/diversity-survey/. [file peerj-cs-06-299-s002.bz2 › sysconf/pubs/web/images/CC-BY-SA.jpg]

File:CC-BY-SA icon.svg - Wikipedia


# File:CC-BY-SA icon.svg

From Wikipedia, the free encyclopedia

Jump to navigation
Jump to search

- File
- File history
- File usage
- Global file usage
- Metadata

Size of this PNG preview of this SVG file: 88 × 31 pixels. Other resolutions: 320 × 113 pixels | 640 × 225 pixels | 800 × 282 pixels | 1,024 × 361 pixels | 1,280 × 451 pixels.

Original file ‎(SVG file, nominally 88 × 31 pixels, file size: 7 KB)

|  |  |
| --- | --- |
|  | This is a file from the Wikimedia Commons. Information from its **description page there** is shown below. Commons is a freely licensed media file repository. You can help. |

## Summary

|  |  |
| --- | --- |
| DescriptionCC-BY-SA icon.svg | **English:** *Creative Commons "Attribution-Share Alike"* license icon.  **Esperanto:** Bildsimbolo de la permesilo *Krea Komunaĵo "Atribuite-Samkondiĉe"*.  **Español:** Ícono de la licencia *Creative Commons "Atribución-Compartir Igual"*  **Français :** Logo de la licence Creative Commons « Paternité-Partage des Conditions Initiales à l'Identique »  **Magyar:** Creative Commons logo: "Nevezd meg!-Így add tovább!" |
| Date | 2008 |
| Source | http://mirrors.creativecommons.org/presskit/buttons/88x31/svg/by-sa.svg |
| Author | Creative Commons |
| Permission (Reusing this file) | See http://creativecommons.org/policies for trademark restrictions. |
| Other versions | - CC-BY-SA 3 icon 88x31.png (PNG version) - CC-BY-SA icon orange.svg (orange version) - Cc-by-sa (1).svg (14KB SVG version) |

|  |
| --- |
| iThe source code of this SVG is valid. |

As stated on http://creativecommons.org/policies use the current SVG on the CC downloads page for downloads. The copy here is intended for MediaWiki InstantCommons.

## Licensing

|  |
| --- |
| Public domainPublic domainfalsefalse |

|  |  |
| --- | --- |
|  | *This image consists only of simple geometric shapes or text. It **does not meet the threshold of originality** needed for copyright protection, and is therefore in the **public domain**. Although it is free of copyright restrictions, this image may still be subject to other restrictions. See WP:PD#Fonts and typefaces or Template talk:PD-textlogo for more information.* |

|  |  |
| --- | --- |
|  | This work includes material that may be **protected as a trademark** in some jurisdictions. If you want to use it, you have to ensure that you have the legal right to do so and that you do not infringe any trademark rights. See our general disclaimer. This tag does not indicate the copyright status of the attached work. A normal copyright tag is still required. See Commons:Licensing. |

|  |  |
| --- | --- |
| Annotations | This image is annotated: **View the annotations at Commons** |

3

1

26

27

88

31

Creative Commons license

36

2

21

19

88

31

Attribution

58

2

22

20

88

31

Share Alike

## File history

Click on a date/time to view the file as it appeared at that time.

|  | Date/Time | Thumbnail | Dimensions | User | Comment |
| --- | --- | --- | --- | --- | --- |
| current | 07:35, 12 September 2012 |  | 88 × 31 (7 KB) | Palosirkka | Scrubbed with http://codedread.com/scour/ |
|  | 15:31, 2 July 2010 |  | 88 × 31 (7 KB) | Fleshgrinder | Optimized and valid SVG |
|  | 16:13, 26 September 2009 |  | 88 × 31 (18 KB) | Petrus Adamus | 88×31 px |
|  | 03:32, 16 April 2009 |  | 120 × 42 (16 KB) | MattWade | {{Information |Description=CC-BY-SA Logo |Source=[http://creativecommons.org/about/downloads/ Creative Commons Logo Downloads] |Date= |Author= w:Creative Commons |Permission=See below |other\_versions= }} [[Category:Creative |

## File usage

More than 100 pages use this file.
The following list shows the first 100 pages that use this file only.
A full list is available.

- 4Licensing Corporation
- America's Next Top Model (season 10)
- Anoka–Champlin Mississippi River Bridge
- Antidepressant
- Arsenic poisoning
- Arthur Naftalin
- Asin of Baekje
- B'nai Abraham Synagogue (Virginia, Minnesota)
- Beltrami County Courthouse
- Belu-Simion Fainaru
- Beop of Baekje
- Betty Brant
- Bhutanese ngultrum
- Biryu of Baekje
- Biyu of Baekje
- Brecknockshire
- Bryan Charnley
- Bunseo of Baekje
- Burbank–Livingston–Griggs House
- Canopy walkway
- Casiville Bullard House
- Chaekgye of Baekje
- Chemical formula
- Chimnyu of Baekje
- Chogo of Baekje
- Cleveland, Minneapolis
- Climate change in New Zealand
- Commerce Building (Saint Paul, Minnesota)
- Constructed wetland
- Cowan, New South Wales
- Creative Commons license
- Daru of Baekje
- Deerwood Auditorium
- Donald Duck
- Dongseong of Baekje
- Driver (video game)
- Economy of Bangladesh
- Education in Australia
- Endion station
- Frederick Spangenberg House
- Gaero of Baekje
- Gaeru of Baekje
- George Bonga
- Geunchogo of Baekje
- Geungusu of Baekje
- Giru of Baekje
- Goi of Baekje
- Goodsell Observatory
- Guardians of the Universe
- Guisin of Baekje
- Gusu of Baekje
- Gye of Baekje
- Heterodont
- Higher School Certificate (New South Wales)
- Hull–Rust–Mahoning Open Pit Iron Mine
- Hye of Baekje
- Industrial Exposition Building
- Inyan Ceyaka Otonwe
- Jeonji of Baekje
- Jinsa of Baekje
- Karl König
- Lasso of Truth
- Lawrence A. and Mary Fournier House
- Leura, New South Wales
- License compatibility
- List of Keeping Up Appearances characters
- List of The Transformers (TV series) characters
- List of challenges in Takeshi's Castle
- List of fictional dogs
- Loan Council
- Magistrates Court of the Australian Capital Territory
- Mallala, South Australia
- Merchants National Bank (Winona, Minnesota)
- Methemoglobin
- Mickey's Diner
- Minneapolis Pioneers and Soldiers Memorial Cemetery
- Minnesota Centennial Showboat
- Minnesota Historical Society
- Mu of Baekje
- Munju of Baekje
- Muryeong of Baekje
- Onjo of Baekje
- Palpitations
- Parade Stadium
- Peter R. Hunt
- Platteville Limestone
- Saban of Baekje
- Saint Joseph's Academy (Saint Paul, Minnesota)
- Samgeun of Baekje
- Saurashtra language
- Scientific Vector Language
- Scrying
- Seong of Baekje
- Spider-Man
- Sugar (software)
- Supreme Court of the Australian Capital Territory
- Swany White Flour Mills
- Tarring and feathering
- Uija of Baekje
- Wideok of Baekje

## Global file usage

The following other wikis use this file:

- Usage on af.wikipedia.org
  - Creative Commons lisensie
- Usage on als.wikipedia.org
  - Datei:Fötzelschnitte.jpg
  - Vorlage:Bild-CC-by-sa/4.0
- Usage on an.wikipedia.org
  - Descusión:Miollo osio
  - Plantilla:Traducción de
  - Descusión:Reflexión (fisica)
  - Descusión:Soraya Sáenz de Santamaría
- Usage on ar.wikipedia.org
  - قالب:لقطة شاشة لويكيبيديا
  - ملف:Save.PNG
  - ملف:Editwiki.PNG
  - ملف:Vote1.PNG
  - ملف:Vote2.PNG
  - ملف:Vote3.PNG
  - ملف:حذف1.jpg
  - ملف:حذف3.jpg
  - ملف:حذف4.jpg
  - ملف:حذف5.jpg
  - ملف:حذف6.jpg
  - ملف:حذف7.jpg
  - ملف:حذف8.jpg
  - ملف:إضافة ترخيص صورة.jpg
  - ملف:واجهة تفضيلات.jpg
  - ملف:الألسنة في فايرفوكس.PNG
  - ملف:الألسنة في فايرفوكس2.PNG
  - ملف:مدفع.jpg
  - رخص المشاع الإبداعي
  - ملف:POP-UPs-ar.png
  - ملف:Arabic Wikipedia basic navigation.jpg
  - ملف:Arwp-mainpage.png
  - ملف:Wikipedia-category-graph.png
  - ملف:سابين بيرغمان بول.jpg
  - ملف:Wilhelm Pieck.jpg
  - ملف:Snowintabuk.jpg
  - ملف:Anabtamap.jpg
  - ملف:Ar wiki 2010 on iPhone.PNG
  - ملف:لقطة شاشة شرف الدين.jpg
  - ملف:لقطة شاشة شرف الدين 2.jpg
  - ملف:PIC1.jpg
  - ملف:ويكيبيديا الأردية.PNG
  - ملف:TIME cover july 31 2000.jpg
  - ملف:Sunwell fish packing pumpable slurry ice.JPG
  - ملف:Cross section nanomesh.jpg
  - ملف:Nanomesh 3D.jpg
  - ملف:Computational Gene.jpg
  - ملف:Diagnosis Cancer.jpg
  - ملف:Therapy cancer.jpg
  - ملف:مستشعر جزيئي.png
  - ملف:AHL DNA 2.GIF
  - ملف:Biochip platform.jpg
  - ملف:Torino-Population.png

View more global usage of this file.

## Metadata

This file contains additional information, probably added from the digital camera or scanner used to create or digitize it.

If the file has been modified from its original state, some details may not fully reflect the modified file.

|  |  |
| --- | --- |
| Short title | Creative Commons “Attribution-Share Alike” license icon |
| Width | 88px |
| Height | 31px |

Retrieved from "https://en.wikipedia.org/wiki/File:CC-BY-SA\_icon.svg"

## Navigation menu

### Personal tools

- Not logged in
- Talk
- Contributions
- Create account
- Log in

### Namespaces

- File
- Talk

### Variants

### Views

- Read
- View on Commons

### More

### Search

### Navigation

- Main page
- Contents
- Featured content
- Current events
- Random article
- Donate to Wikipedia
- Wikipedia store

### Interaction

- Help
- About Wikipedia
- Community portal
- Recent changes
- Contact page

### Tools

- What links here
- Upload file
- Special pages
- Page information

### Languages

- Privacy policy
- About Wikipedia
- Disclaimers
- Contact Wikipedia
- Developers
- Cookie statement
- Mobile view
