# Supplementary figures and images for: A survey of accepted authors in computer systems conferences
Source: PeerJ Comput Sci. 2020 Sep 28;6:e299. doi: 10.7717/peerj-cs.299 (PMC7924675; doi:10.7717/peerj-cs.299)

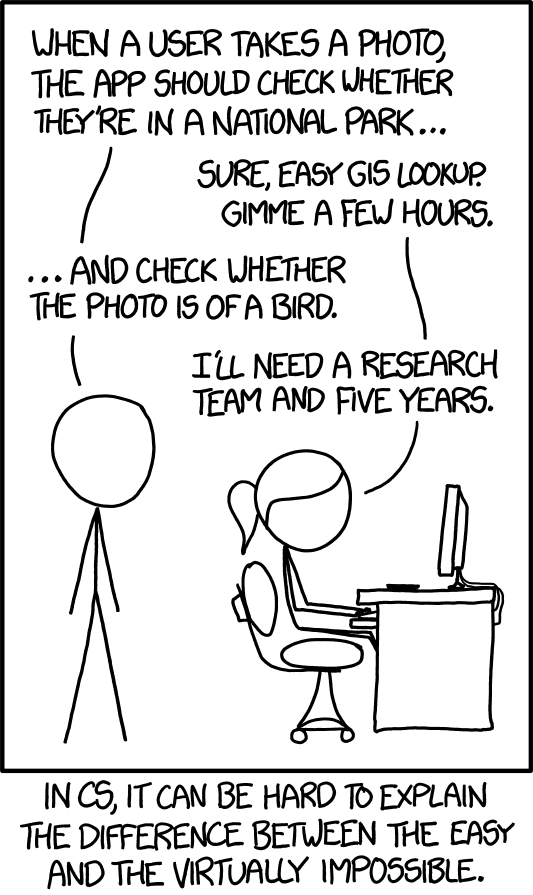

Supplement: Supplemental Information 2 — This is a snapshot of the github repository that includes the data and source code required to reproduce this paper (except for confidential survey data). The snapshot represents commit 6663a253f1ac4dc351a78ccc74c0de80c7cc06ad of http://github.com/eitanf/sysconf. The most pertinent article files are under pubs/diversity-survey/. [file peerj-cs-06-299-s002.bz2 › sysconf/pubs/web/images/tasks_2x.png]

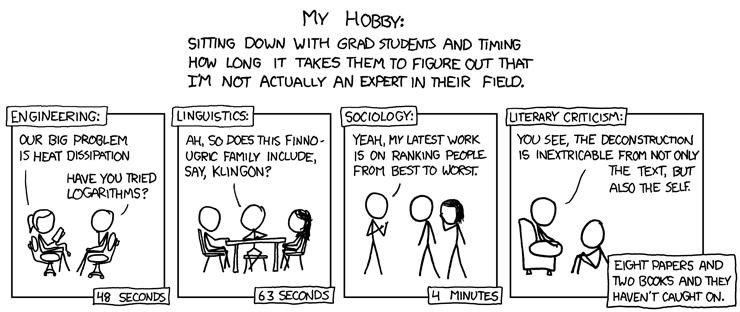

Supplement: Supplemental Information 2 — This is a snapshot of the github repository that includes the data and source code required to reproduce this paper (except for confidential survey data). The snapshot represents commit 6663a253f1ac4dc351a78ccc74c0de80c7cc06ad of http://github.com/eitanf/sysconf. The most pertinent article files are under pubs/diversity-survey/. [file peerj-cs-06-299-s002.bz2 › sysconf/pubs/web/images/impostor.png]

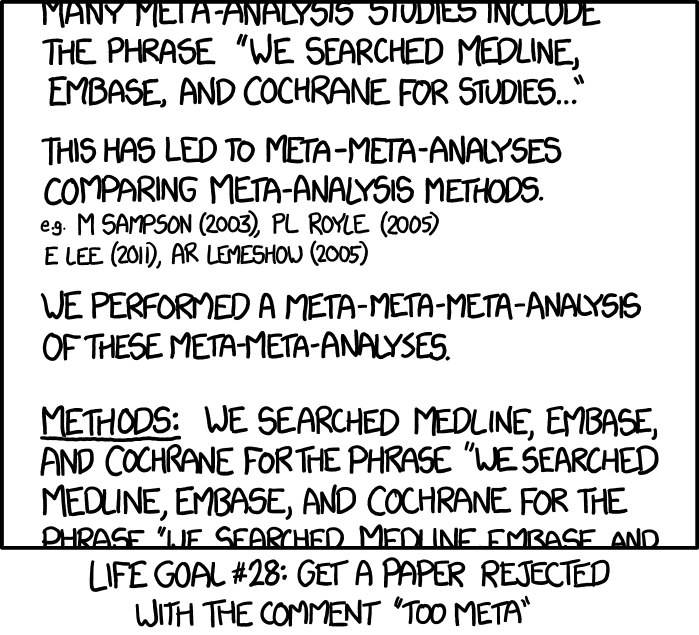

Supplement: Supplemental Information 2 — This is a snapshot of the github repository that includes the data and source code required to reproduce this paper (except for confidential survey data). The snapshot represents commit 6663a253f1ac4dc351a78ccc74c0de80c7cc06ad of http://github.com/eitanf/sysconf. The most pertinent article files are under pubs/diversity-survey/. [file peerj-cs-06-299-s002.bz2 › sysconf/pubs/web/images/meta-analysis_2x.png]

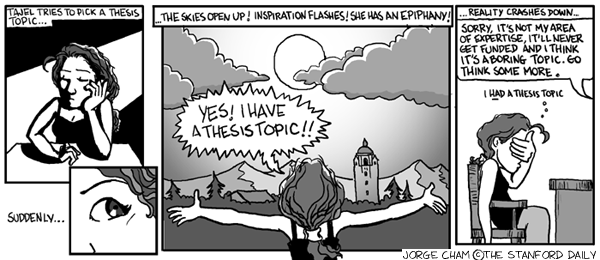

Supplement: Supplemental Information 2 — This is a snapshot of the github repository that includes the data and source code required to reproduce this paper (except for confidential survey data). The snapshot represents commit 6663a253f1ac4dc351a78ccc74c0de80c7cc06ad of http://github.com/eitanf/sysconf. The most pertinent article files are under pubs/diversity-survey/. [file peerj-cs-06-299-s002.bz2 › sysconf/pubs/web/images/phd100998s.gif]

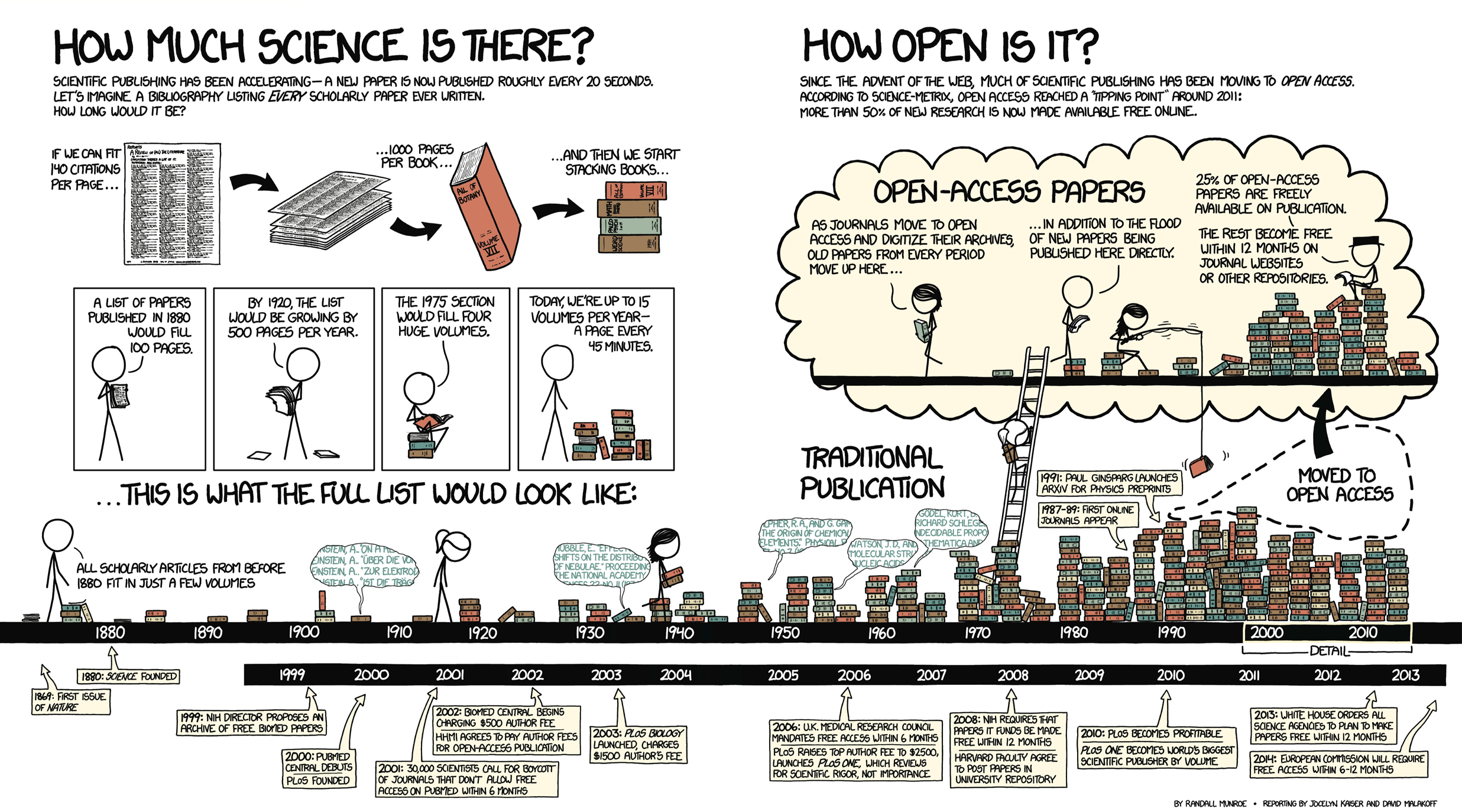

Supplement: Supplemental Information 2 — This is a snapshot of the github repository that includes the data and source code required to reproduce this paper (except for confidential survey data). The snapshot represents commit 6663a253f1ac4dc351a78ccc74c0de80c7cc06ad of http://github.com/eitanf/sysconf. The most pertinent article files are under pubs/diversity-survey/. [file peerj-cs-06-299-s002.bz2 › sysconf/pubs/web/images/infographic.jpg]

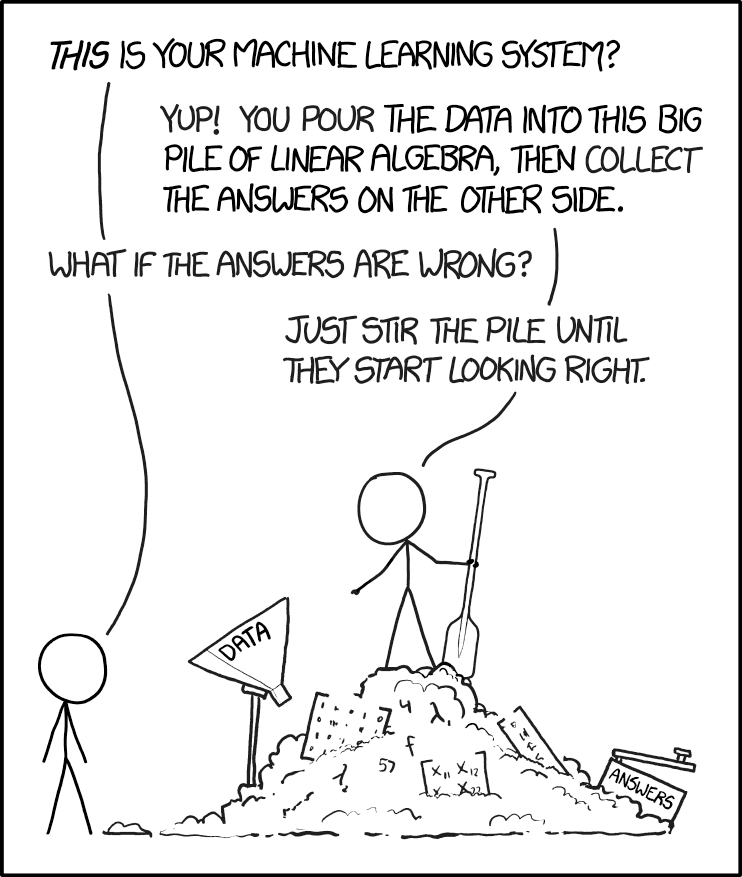

Supplement: Supplemental Information 2 — This is a snapshot of the github repository that includes the data and source code required to reproduce this paper (except for confidential survey data). The snapshot represents commit 6663a253f1ac4dc351a78ccc74c0de80c7cc06ad of http://github.com/eitanf/sysconf. The most pertinent article files are under pubs/diversity-survey/. [file peerj-cs-06-299-s002.bz2 › sysconf/pubs/web/images/machine_learning_2x.png]

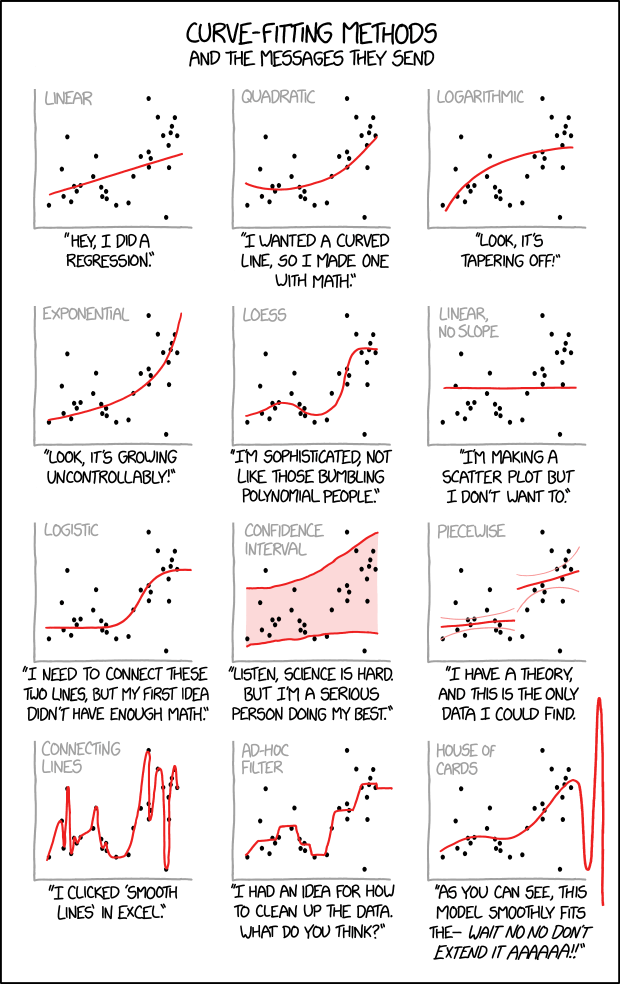

Supplement: Supplemental Information 2 — This is a snapshot of the github repository that includes the data and source code required to reproduce this paper (except for confidential survey data). The snapshot represents commit 6663a253f1ac4dc351a78ccc74c0de80c7cc06ad of http://github.com/eitanf/sysconf. The most pertinent article files are under pubs/diversity-survey/. [file peerj-cs-06-299-s002.bz2 › sysconf/pubs/web/images/curve_fitting.png]

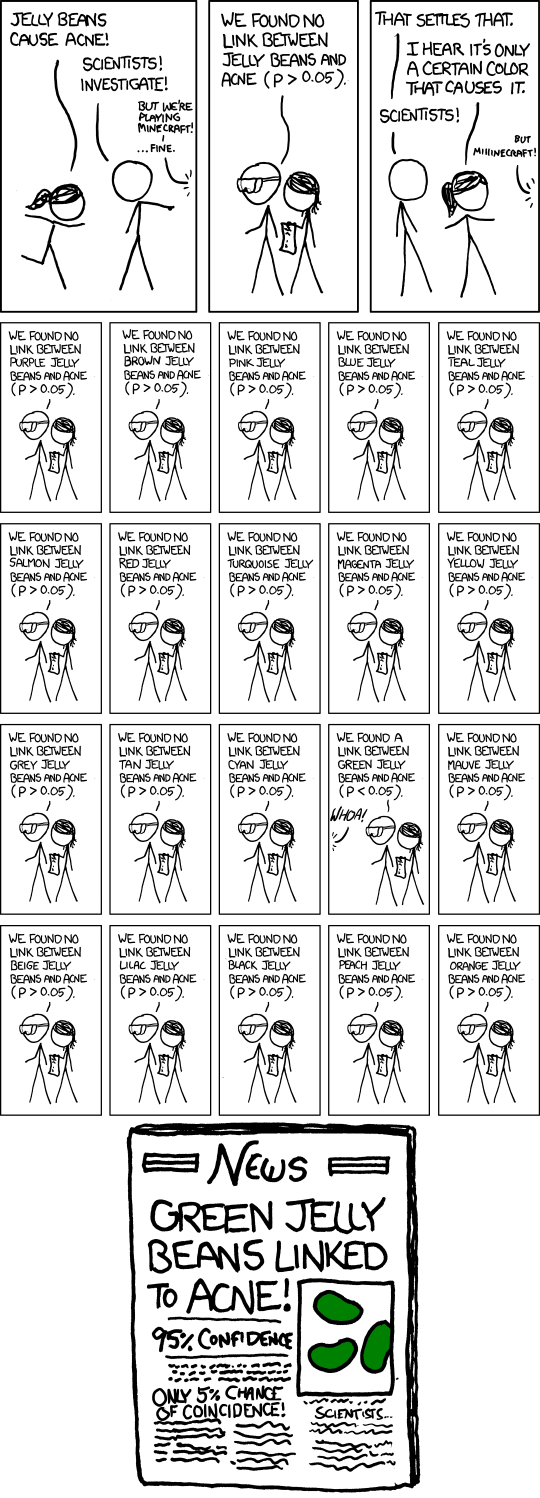

Supplement: Supplemental Information 2 — This is a snapshot of the github repository that includes the data and source code required to reproduce this paper (except for confidential survey data). The snapshot represents commit 6663a253f1ac4dc351a78ccc74c0de80c7cc06ad of http://github.com/eitanf/sysconf. The most pertinent article files are under pubs/diversity-survey/. [file peerj-cs-06-299-s002.bz2 › sysconf/pubs/web/images/significant.png]

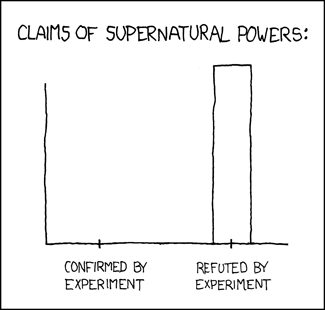

Supplement: Supplemental Information 2 — This is a snapshot of the github repository that includes the data and source code required to reproduce this paper (except for confidential survey data). The snapshot represents commit 6663a253f1ac4dc351a78ccc74c0de80c7cc06ad of http://github.com/eitanf/sysconf. The most pertinent article files are under pubs/diversity-survey/. [file peerj-cs-06-299-s002.bz2 › sysconf/pubs/web/images/the_data_so_far.png]

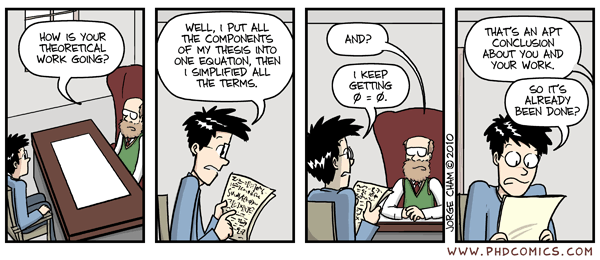

Supplement: Supplemental Information 2 — This is a snapshot of the github repository that includes the data and source code required to reproduce this paper (except for confidential survey data). The snapshot represents commit 6663a253f1ac4dc351a78ccc74c0de80c7cc06ad of http://github.com/eitanf/sysconf. The most pertinent article files are under pubs/diversity-survey/. [file peerj-cs-06-299-s002.bz2 › sysconf/pubs/web/images/phd110110s.gif]

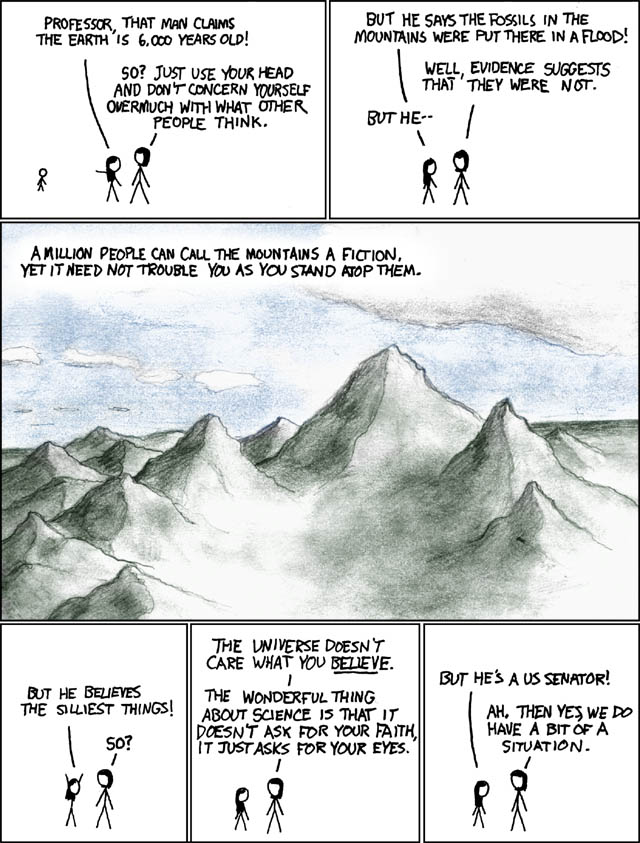

Supplement: Supplemental Information 2 — This is a snapshot of the github repository that includes the data and source code required to reproduce this paper (except for confidential survey data). The snapshot represents commit 6663a253f1ac4dc351a78ccc74c0de80c7cc06ad of http://github.com/eitanf/sysconf. The most pertinent article files are under pubs/diversity-survey/. [file peerj-cs-06-299-s002.bz2 › sysconf/pubs/web/images/beliefs.jpg]

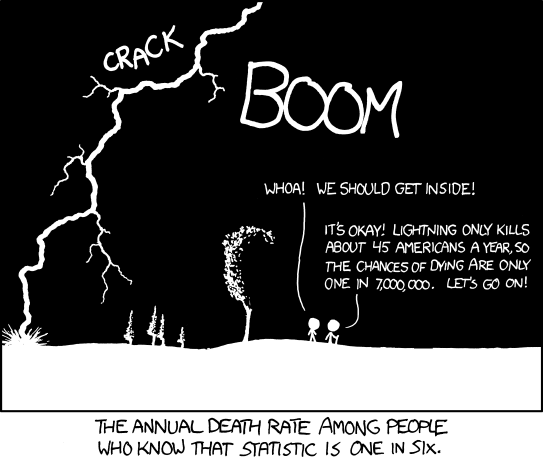

Supplement: Supplemental Information 2 — This is a snapshot of the github repository that includes the data and source code required to reproduce this paper (except for confidential survey data). The snapshot represents commit 6663a253f1ac4dc351a78ccc74c0de80c7cc06ad of http://github.com/eitanf/sysconf. The most pertinent article files are under pubs/diversity-survey/. [file peerj-cs-06-299-s002.bz2 › sysconf/pubs/web/images/conditional_risk.png]

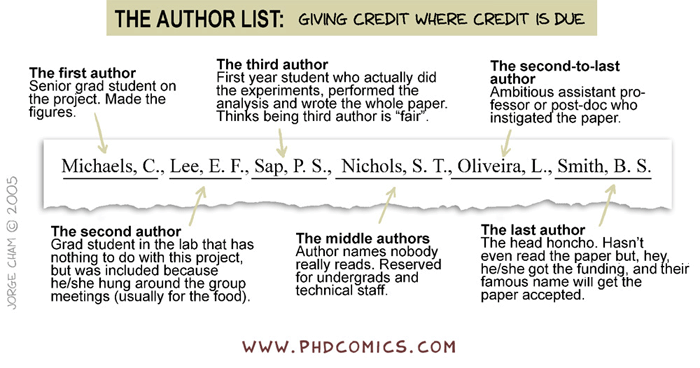

Supplement: Supplemental Information 2 — This is a snapshot of the github repository that includes the data and source code required to reproduce this paper (except for confidential survey data). The snapshot represents commit 6663a253f1ac4dc351a78ccc74c0de80c7cc06ad of http://github.com/eitanf/sysconf. The most pertinent article files are under pubs/diversity-survey/. [file peerj-cs-06-299-s002.bz2 › sysconf/pubs/web/images/phd031305s.gif]

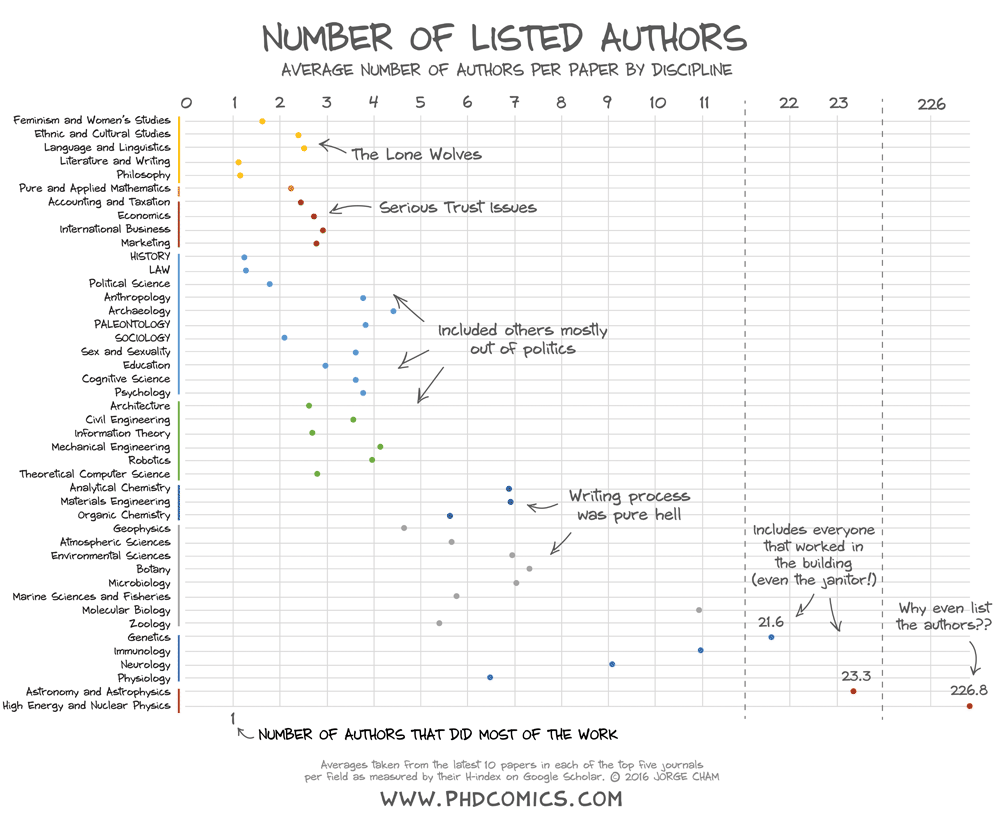

Supplement: Supplemental Information 2 — This is a snapshot of the github repository that includes the data and source code required to reproduce this paper (except for confidential survey data). The snapshot represents commit 6663a253f1ac4dc351a78ccc74c0de80c7cc06ad of http://github.com/eitanf/sysconf. The most pertinent article files are under pubs/diversity-survey/. [file peerj-cs-06-299-s002.bz2 › sysconf/pubs/web/images/phd120916s.gif]

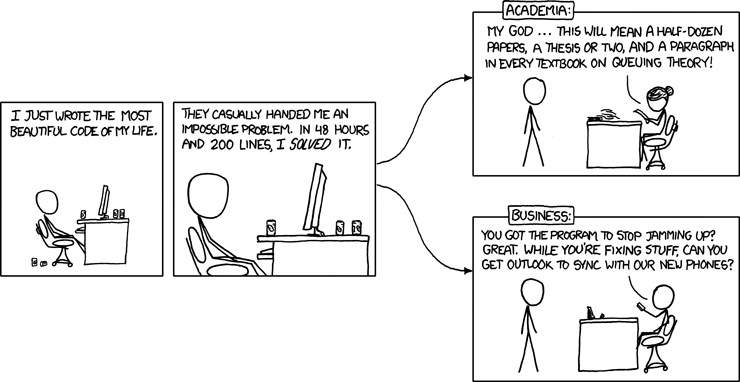

Supplement: Supplemental Information 2 — This is a snapshot of the github repository that includes the data and source code required to reproduce this paper (except for confidential survey data). The snapshot represents commit 6663a253f1ac4dc351a78ccc74c0de80c7cc06ad of http://github.com/eitanf/sysconf. The most pertinent article files are under pubs/diversity-survey/. [file peerj-cs-06-299-s002.bz2 › sysconf/pubs/web/images/academia_vs_business.png]

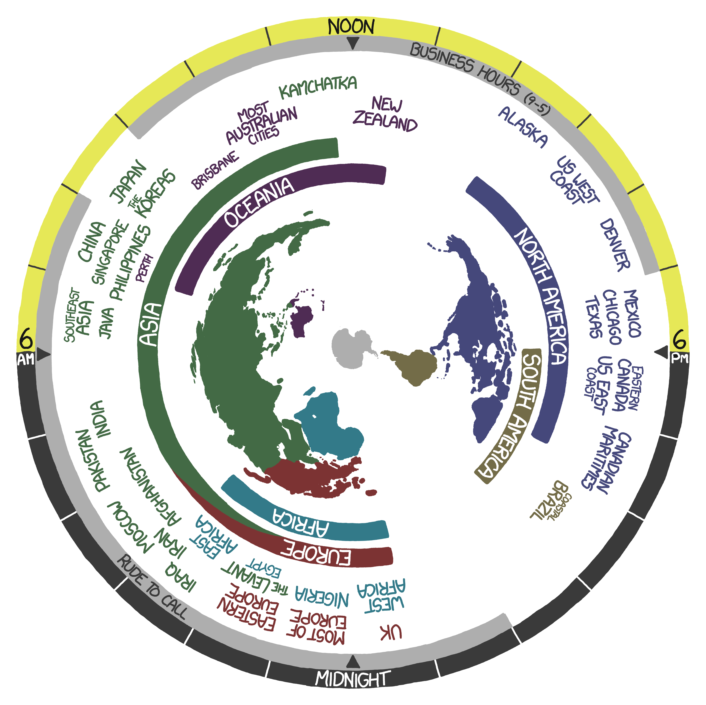

Supplement: Supplemental Information 2 — This is a snapshot of the github repository that includes the data and source code required to reproduce this paper (except for confidential survey data). The snapshot represents commit 6663a253f1ac4dc351a78ccc74c0de80c7cc06ad of http://github.com/eitanf/sysconf. The most pertinent article files are under pubs/diversity-survey/. [file peerj-cs-06-299-s002.bz2 › sysconf/pubs/web/images/11h30m.png]

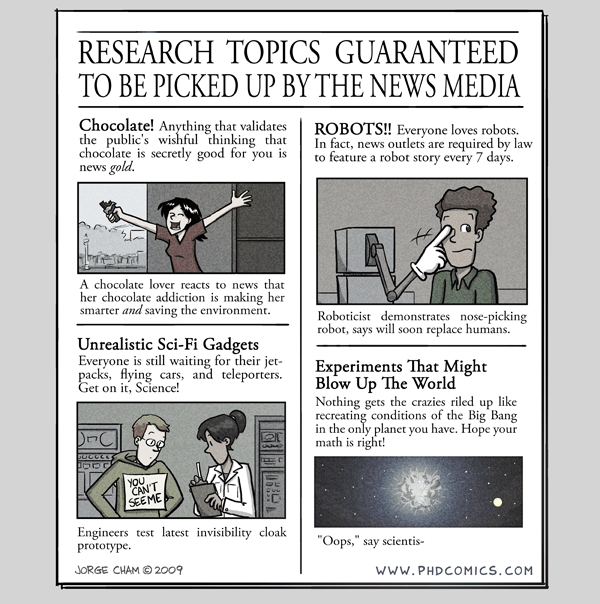

Supplement: Supplemental Information 2 — This is a snapshot of the github repository that includes the data and source code required to reproduce this paper (except for confidential survey data). The snapshot represents commit 6663a253f1ac4dc351a78ccc74c0de80c7cc06ad of http://github.com/eitanf/sysconf. The most pertinent article files are under pubs/diversity-survey/. [file peerj-cs-06-299-s002.bz2 › sysconf/pubs/web/images/phd052009s.gif]

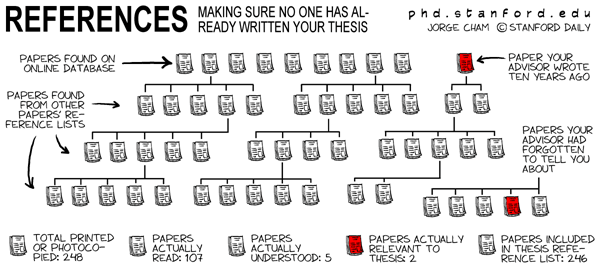

Supplement: Supplemental Information 2 — This is a snapshot of the github repository that includes the data and source code required to reproduce this paper (except for confidential survey data). The snapshot represents commit 6663a253f1ac4dc351a78ccc74c0de80c7cc06ad of http://github.com/eitanf/sysconf. The most pertinent article files are under pubs/diversity-survey/. [file peerj-cs-06-299-s002.bz2 › sysconf/pubs/web/images/phd022702s.gif]

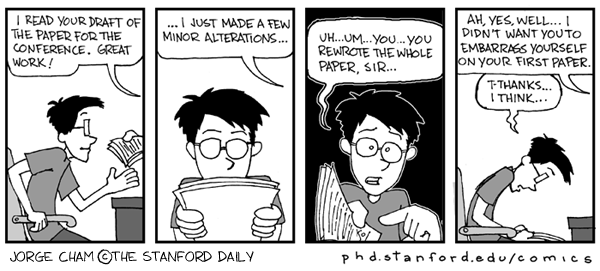

Supplement: Supplemental Information 2 — This is a snapshot of the github repository that includes the data and source code required to reproduce this paper (except for confidential survey data). The snapshot represents commit 6663a253f1ac4dc351a78ccc74c0de80c7cc06ad of http://github.com/eitanf/sysconf. The most pertinent article files are under pubs/diversity-survey/. [file peerj-cs-06-299-s002.bz2 › sysconf/pubs/web/images/phd010500s.gif]

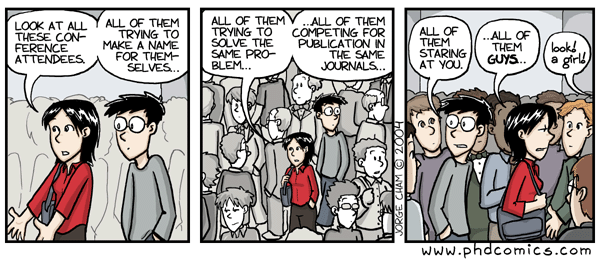

Supplement: Supplemental Information 2 — This is a snapshot of the github repository that includes the data and source code required to reproduce this paper (except for confidential survey data). The snapshot represents commit 6663a253f1ac4dc351a78ccc74c0de80c7cc06ad of http://github.com/eitanf/sysconf. The most pertinent article files are under pubs/diversity-survey/. [file peerj-cs-06-299-s002.bz2 › sysconf/pubs/web/images/phd081604s.gif]

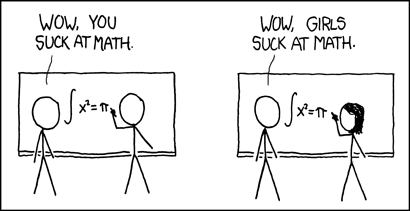

Supplement: Supplemental Information 2 — This is a snapshot of the github repository that includes the data and source code required to reproduce this paper (except for confidential survey data). The snapshot represents commit 6663a253f1ac4dc351a78ccc74c0de80c7cc06ad of http://github.com/eitanf/sysconf. The most pertinent article files are under pubs/diversity-survey/. [file peerj-cs-06-299-s002.bz2 › sysconf/pubs/web/images/how_it_works.png]

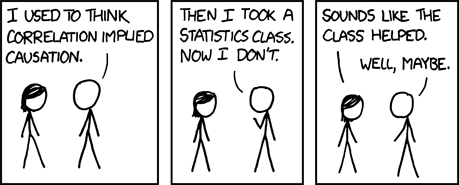

Supplement: Supplemental Information 2 — This is a snapshot of the github repository that includes the data and source code required to reproduce this paper (except for confidential survey data). The snapshot represents commit 6663a253f1ac4dc351a78ccc74c0de80c7cc06ad of http://github.com/eitanf/sysconf. The most pertinent article files are under pubs/diversity-survey/. [file peerj-cs-06-299-s002.bz2 › sysconf/pubs/web/images/correlation.png]
