# Supplementary material for: A survey of accepted authors in computer systems conferences
Source: PeerJ Comput Sci. 2020 Sep 28;6:e299. doi: 10.7717/peerj-cs.299 (PMC7924675; doi:10.7717/peerj-cs.299)
Supplement: Supplemental Information 2 — This is a snapshot of the github repository that includes the data and source code required to reproduce this paper (except for confidential survey data). The snapshot represents commit 6663a253f1ac4dc351a78ccc74c0de80c7cc06ad of http://github.com/eitanf/sysconf. The most pertinent article files are under pubs/diversity-survey/. [file peerj-cs-06-299-s002.bz2 › sysconf/data/cfp/PACT.pdf]

PACT2017

THE 26TH INTERNATIONAL CONFERENCE ON PARALLEL ARCHITECTURES AND COMPILATION TECHNIQUES

SEPTEMBER 9-13, PORTLAND, OR

Call for Papers

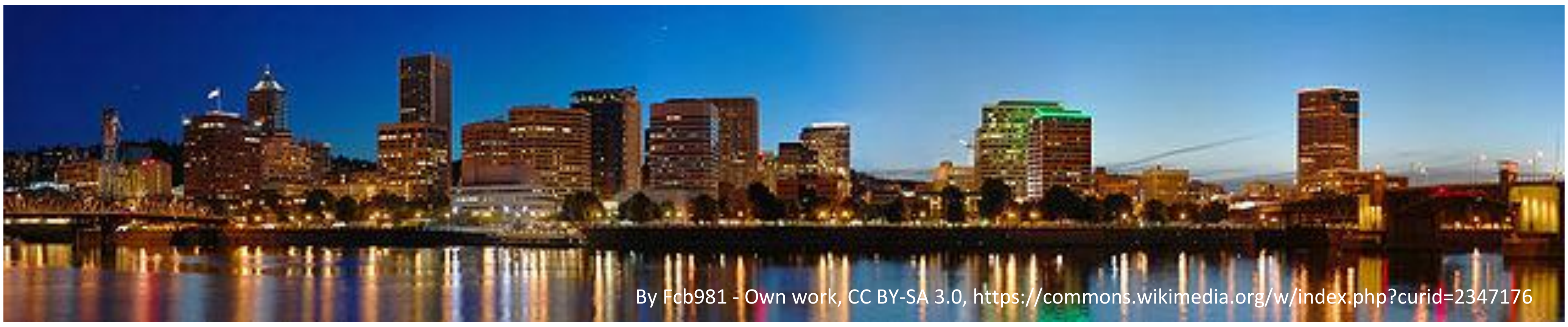
By Fcb981 - Own work, CC BY-SA 3.0, https://commons.wikimedia.org/w/index.php?curid=2347176

About PACT

The purpose of PACT 2017 is to bring together researchers from architecture, compilers, applications and languages to present and discuss innovative research of common interest.

PACT started as a Data Flow Workshop in conjunction with ISCA 1989 but quickly evolved into a unique venue at the intersection of classical parallel architecture and compilers.

Recently, PACT widened its scope to include insights useful for the design of machines and compilers from applications such as, but not limited to, machine learning, data analytics and computational biology.

PACT solicits novel papers, workshops, tutorials, and entries to an ACM student research competition on a broad range of topics that include, but are not limited to:

- Parallel architectures and computational models
- Compilers and tools for parallel computer systems
- Multicore, multithreaded, superscalar, and VLIW architectures
- Compiler/hardware support for dealing with memory latencies
- Support for correctness in concurrent hardware and software
- Reconfigurable parallel computing
- Dynamic translation and optimization
- I/O issues in parallel computing and their relation to applications
- Parallel programming languages, algorithms and applications
- Middleware and run time system support for parallel computing
- Application-specific parallel systems
- Applications and experimental systems studies of parallel processing
- Relevant aspects of distributed computing and mobile computing
- Heterogeneous systems using various types of accelerators
- Insights from modern parallel applications such as, but not limited to, machine learning, data analytics, and computational biology for the design of parallel architectures and compilers

Submissions

Submitted papers will be evaluated on technical merits and clarity of presentation. Papers must contain sufficient information and be organized in such a way that their technical contribution and significance can be understood by a wide audience of computer scientists.

Submitted papers must be original material that has not been previously published in another conference or journal, nor is currently under review by another conference or journal. Papers are to be submitted for double-blind review. This means that author names as well as hints of identity are to be removed from the submitted paper.

Authors of accepted papers will be invited to formally submit their supporting materials to the Artifact Evaluation Committee. The task of this committee is to assess how the artifacts support the work described in the papers. Submission is voluntary. Papers that go through the artifact evaluation process successfully will receive a seal of approval which will be printed on the first page of the papers in the proceedings. Authors of accepted papers are encouraged (but not obliged) to make these materials publicly available upon publication of the proceedings, by including them as "source materials" in the ACM Digital Library.

AUTHORS TAKE NOTE: The official publication date is the date the proceedings are made available in the ACM Digital Library or IEEE Xplore. This date may be up to two weeks prior to the first day of the conference. The official publication date affects the deadline for any patent filings related to published work. (For those rare conferences whose proceedings are published in the ACM Digital Library or IEEE Xplore after the conference is over, the official publication date remains the first day of the conference.)

CONTACTS

General Chair
Ravi Iyer, Intel

Program Chair
David Padua, University of Illinois

IMPORTANT DATES

Paper Deadline:
March 14, 2017

Author Response Period:
May 3-6, 2017

Author Notification:
May 24, 2017

Camera Ready Final Papers:
July 19, 2017

Association for Computing Machinery

IEEE computer society

ifip

For more information:

http://pactconf.org/
